# The value of MRI for differentiating benign from malignant sex cord-stromal tumors of the ovary: emphasis on diffusion-weighted MR imaging

**DOI:** 10.1186/s13048-018-0444-6

**Published:** 2018-08-30

**Authors:** Shu-Hui Zhao, Hai-Ming Li, Jin-Wei Qiang, Deng-Bin Wang, Hua Fan

**Affiliations:** 1 Department of Radiology, Xinhua Hospital affiliated to Shanghai Jiaotong University School of Medicine, 1665 Kongjiang Road, Shanghai, 200092 China; 2 Department of Radiology, Jinshan Hospital, Fudan University, 1508 Longhang Road, Shanghai, 201508 China

**Keywords:** Ovary, Sex cord-stromal tumor, Diffusion-weighted MR imaging

## Abstract

**Background:**

To investigate MRI for differentiating benign from malignant sex cord-stromal tumors of the ovary (SCSTs) emphasizing on the value of diffusion-weighted (DW) magnetic resonance (MR) imaging.

**Methods:**

This retrospective study included 29 benign SCSTs in 28 patients and 13 malignant SCSTs in 13 patients. DW imaging as well as conventional MR imaging was performed. Signal intensity on DW imaging was assessed and apparent diffusion coefficient (ADC) value was measured. In addition, T2 signal intensity and contrast enhancement pattern were also assessed and compared between benign and malignant SCSTs.

**Results:**

Both of the T2 hypointensity and mild enhancement were specific to benign SCSTs. The majority of malignant SCSTs showed high signal intensity on DW imaging, whereas most benign SCSTs showed low or moderate signal intensity (*p* = 0.000). Fibromas were the tumors with the lowest observed ADC value (0.470 × 10^− 3^ mm^2^/s). Sclerosing stromal tumors were the tumors with the highest observed ADC value (2.291 × 10^− 3^ mm^2^/s). ADC value of solid component was significantly lower in malignant SCSTs (0.825 ± 0.129 × 10^− 3^ mm^2^/s) than in benign SCSTs (1.343 ± 0.528 × 10^− 3^ mm^2^/s) when fibromas were excluded (*p* = 0.024). T2, DCE and DW imaging has a limited value on the differential diagnosis of the benign and malignant SCSTs with an accuracy of 69.0%,71.4% and 78.1% respectively. Combination of T2, DCE and DW imaging permitted the distinction with an accuracy of 88.0%.

**Conclusions:**

It is more helpful for distinction of the benign and malignant SCSTs by combining of T2, DCE and DW imaging than using each of the three sequences independently.

## Background

Ovarian sex cord-stromal tumors (SCSTs) are a group of neoplasm arising from stromal cells and primitive sex cord, accounting for 8% of all ovarian tumors [1–3]. They can affect women of any age [4, 5]. Patients with SCSTs often present virilization syndromes or hyperestrogenic manifestations associated with steroid hormone overproduction [6, 7]. According to World Health Organization (WHO) classification of the ovarian tumors (2014), SCSTs are divided into three clinicopathologic subcategories as pure stromal tumors, pure sex cord tumors and mixed sex cord-stromal tumors [3].

Morphologically, SCSTs often present as a solid mass [8–13]. On conventional MRI, some features have been found to distinguish SCSTs: Fibromas, known as the most common type of SCSTs, are characterized by very low signal intensity on T2WI and delayed mild enhancement. Sclerosing stromal tumor shows low signal intensity in peripheral area and moderate to high signal intensity in central area on T2WI with intense enhancement. Granulosa cell tumor is multiloculated cystic mass with thickened wall or mixed cystic and solid mass [9, 10]. Sertoli-Leydig cell tumor shows as a well-defined solid mass with numerous intratumoral cysts [1]. However, it is still a challenge for radiologists to differentiate benign from malignant SCSTs based on conventional MRI preoperatively [1–3]. Diffusion-weighted MR imaging has been proved to be helpful in characterizing epithelial tumors of the ovary. To our knowledge, a comprehensive study on diffusion-weighted MR imaging features specific to SCSTs have not been reported [8]. In this study, we investigate diffusion-weighted imaging of 42 ovarian sex cord-stromal tumors in 41 patients (29 benign SCSTs in 28 patients and 13 malignant SCSTs in 13 patients) to differentiate benign from malignant sex cord-stromal tumors of the ovary. T2 signal intensity and contrast enhancement pattern were also assessed and compared between benign and malignant SCSTs.

## Methods

### Patient population

The institutional review board of our hospitals approved this retrospective study, and informed consent was obtained from each patient for academic use of their clinical data. Patients with suspected ovarian tumors were enrolled in an ovarian tumor MRI study project from January 2010 to November 2016 at Xinhua Hospital affiliated to Shanghai Jiaotong University School of Medicine and Obstetrics & Gynecology Hospital of Fudan University. We found a total of 41 patients with 42 primary ovarian sex cord-stromal tumors (29 benign SCSTs in 28 patients and 13 malignant SCSTs in 13 patients) (Table 1). Patients’ ages ranged from 24 to 81 years (mean, 51.6 ± 16.3 years) in the benign group and 22–70 years (mean, 41.0 ± 16.2 years) in the malignant group, with no significant difference (*p* = 0.795) (Table 1). Patients presented with abdominal swelling, irregular menstruation cycle, postmenstrual vaginal bleeding, virilization or no symptom. All patients underwent surgery within 1 week after completing the MRI scan.

**Table 1:** Data of benign and malignant ovarian sex cord-stromal tumors

	Number (tumors/patients)	Patient age (years)	Mean size (cm)	Mean ADC value (×10^−3^ mm^2^/s)
Benign	(28/29)#			
Purely stromal tumors				
Fibroma	10	50 (24–81)	6.1	0.470 ± 0.389
Cellular fibroma	2	64 (59–68)	5.2	0.794 ± 0.159
Thecoma	7	64 (48–86)	10.2	1.207 ± 0.350
Fibrothecoma	6	55 (41–70)	11.9	1.150 ± 0.275
Sclerosing stromal tumor	4	28 (26–30)	5.2	2.291 ± 0.423
Malignant	(13/13)			
Purely sex cord tumors				
Granulosa cell tumor	7	41 (27–55)	13.2	0.694 ± 0.111
Sex cord tumor with annular tubule	1	26	2.4	0.975
Mixed Sex cord-stromal tumors				
Sertoli-leydig tumor	4	41 (22–56)	13.0	1.009 ± 0.151
Granulosathecoma	1	70	8.0	0.773

# One of the 28 patients in benign group had bilateral ovarian sex cord-stromal tumors (fibroma and fibrothecoma)

### MR technique

MRI was performed with a 1.5 Tesla (T) MR superconductor unit (Avanto, Siemens, Erlangen, Germany). A pelvic phased-array coil was used in each case. The patients lay in the supine position and breathed normally. The scanning range was from the inferior pubic symphysis to the renal hilum and was extended beyond the dome of tumor in the cases with huge masses.

First, the unenhanced conventional sequences were obtained as follows: axial T1-weighted imaging (T1WI) spin-echo (repetition time/echo time [TR/TE], 340 ms/10 ms), T2-weighted imaging (T2WI) turbo spin-echo with and without fat saturation (TR/TE, 8000 ms/83 ms and 4000 ms/98 ms), and sagittal and coronal T2WI turbo spin-echo (TR/TE, 4000 ms/98 ms).

Second, axial DW imaging was obtained using echo planar imaging sequence. The scanning parameters were as follows: TR/TE, 3200 ms/87 ms; diffusion gradient b factors, 0 and 1000 s/mm2; matrix, 128 × 128; field of view, 238 mm × 280 mm; ST/IG, 5.0 mm/1.5 mm; excitations, 4; acquisition time, 2 min 46 s.

Finally, the contrast enhanced T1WI flash 2D with fat saturation (TR/TE, 196 ms/2.9 ms) was performed in the axial, sagittal, and coronal planes at 30s, 60s and 90s after the intravenous administration of gadopentetate dimeglumine (Gd-DTPA, 0.1 mmol/kg of body weight, Magnevist; Bayer Schering, Guangzhou, China) injected at a rate of 2–3 ml/s. The scanning parameters were as follows: 5-mm slice thickness, 1.5-mm gap, 256 × 256 matrix, 20–25 cm × 34 cm field of view, and four excitations.

### Image analysis

MR images were analyzed by S.H.Z. and H.M.L., with 10 and 8 years of experience in gynecological imaging, respectively. Their interpretations were arrived at by consensus.

1. The signal intensity (SI) of the tumor on T2W imaging was qualitatively evaluated. It was classified as hypointensity (similar signal to the muscle), isointensity (similar signal to the myometrium) and hyperintensity (higher signal than the myometrium).
2. The contrast enhancement of tumor was also qualitatively evaluated on images which acquired at 90s after administration of contrast medium. It was classified as and mild (similar to or weak than the muscles), moderate (between the muscles and the myometrium) and intense (similar to or stronger than the myometrium) enhancement.
3. The signal intensity (SI) of tumor on DW imaging was classified as low (lower than small intestine), moderate (similar to small intestine) or high (similar to the SI of nerve root). On ADC maps, a circular region of interest (ROI) of at least 1 cm^2^ was placed at targeted areas in the solid components of tumor, by referring to conventional MR images including contrast-enhanced images. ROI should avoid areas such as hemorrhage, necrosis, major vascular structures, and artefacts. At least three measurements were obtained and averaged.
4. The degeneration (edema, hemorrhage, and demarcated cysts) of SCSTs was determined by analyzing unenhanced conventional MR images (T1WI, T2WI, T2FS), DW images and the contrast enhanced T1FS images.

### Statistical analysis

Statistical analysis was performed using SPSS 23.0 for Mac (IBM Inc., New York, USA). Wilcoxon rank sum test was used to compare the differences of DW imaging SI between benign SCSTs and malignant SCSTs. One-way ANOVA was performed to compare the ADC values between benign SCSTs and malignant SCSTs. Wilcoxon rank sum test and chi-square test were used to compare the differences of T2 signal intensity and contrast enhancement pattern between benign SCSTs and malignant SCSTs. A *p* value less than 0.05 was regarded as statistically significant.

## Results

Forty-two primary SCSTs were comprised of 29 benign lesions and 13 malignant lesions. The majority of malignant lesions were on stage I (FIGO) except for two lesions (granulosa cell tumors) on stage II (FIGO).

**Fig. 1:**
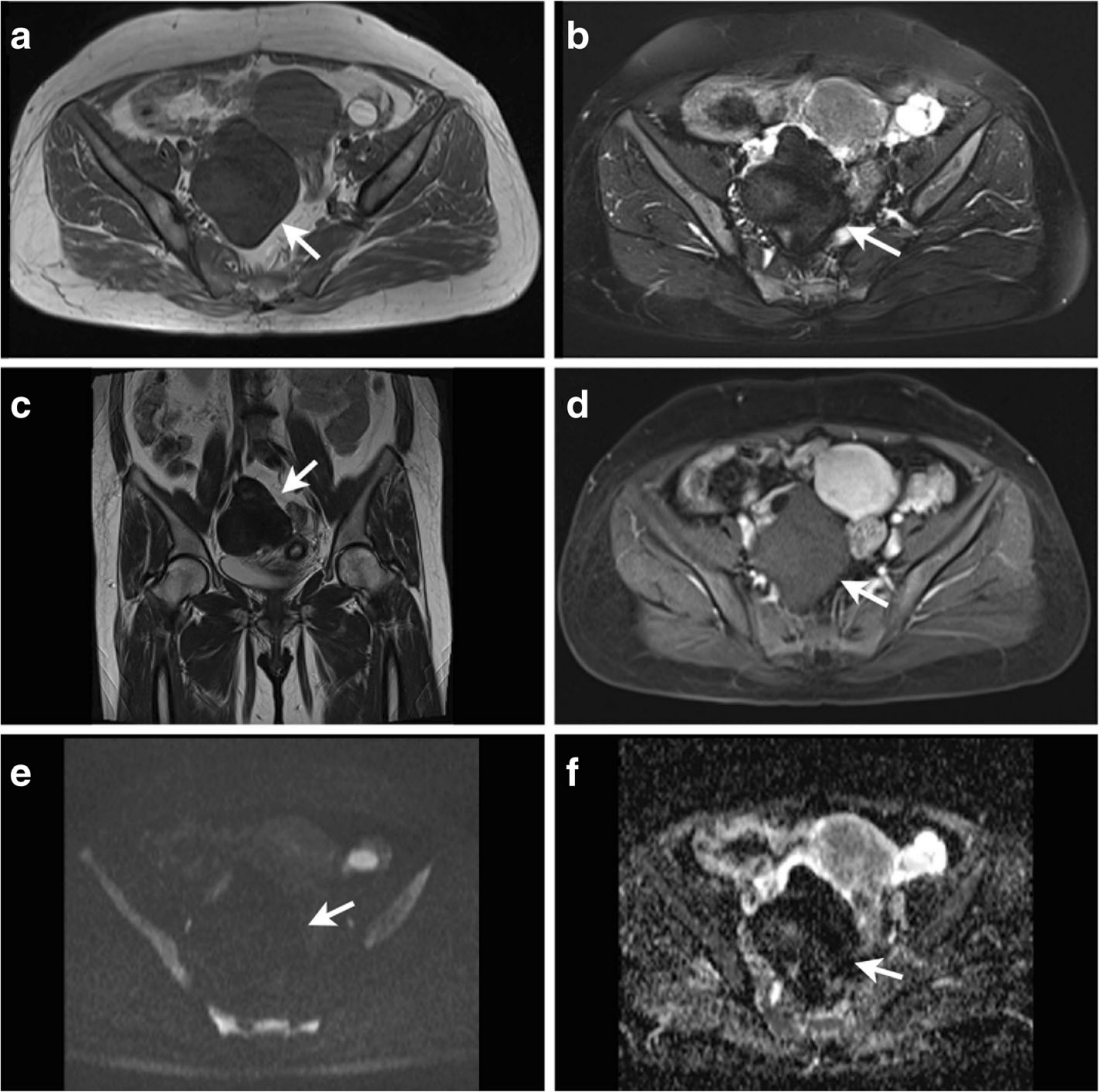
A 47-year-old women with a fibroma on the right ovary(arrows). Axial T1-weighted image (**a**) shows an oval mass of low signal intensity. Axial T2-weighted with fat suppression image (**b**) and coronal T2-weighted image (**c**) shows the mass was homogenous low signal intensity. T1-weighted contrast enhanced images (**d**) shows the mass was slightly enhanced. The mass shows low signal intensity on DW imaging (**e**) and has a significant low ADC value of 0.132 × 10^− 3^ mm^2^/s (**f**)

**Fig. 2:**
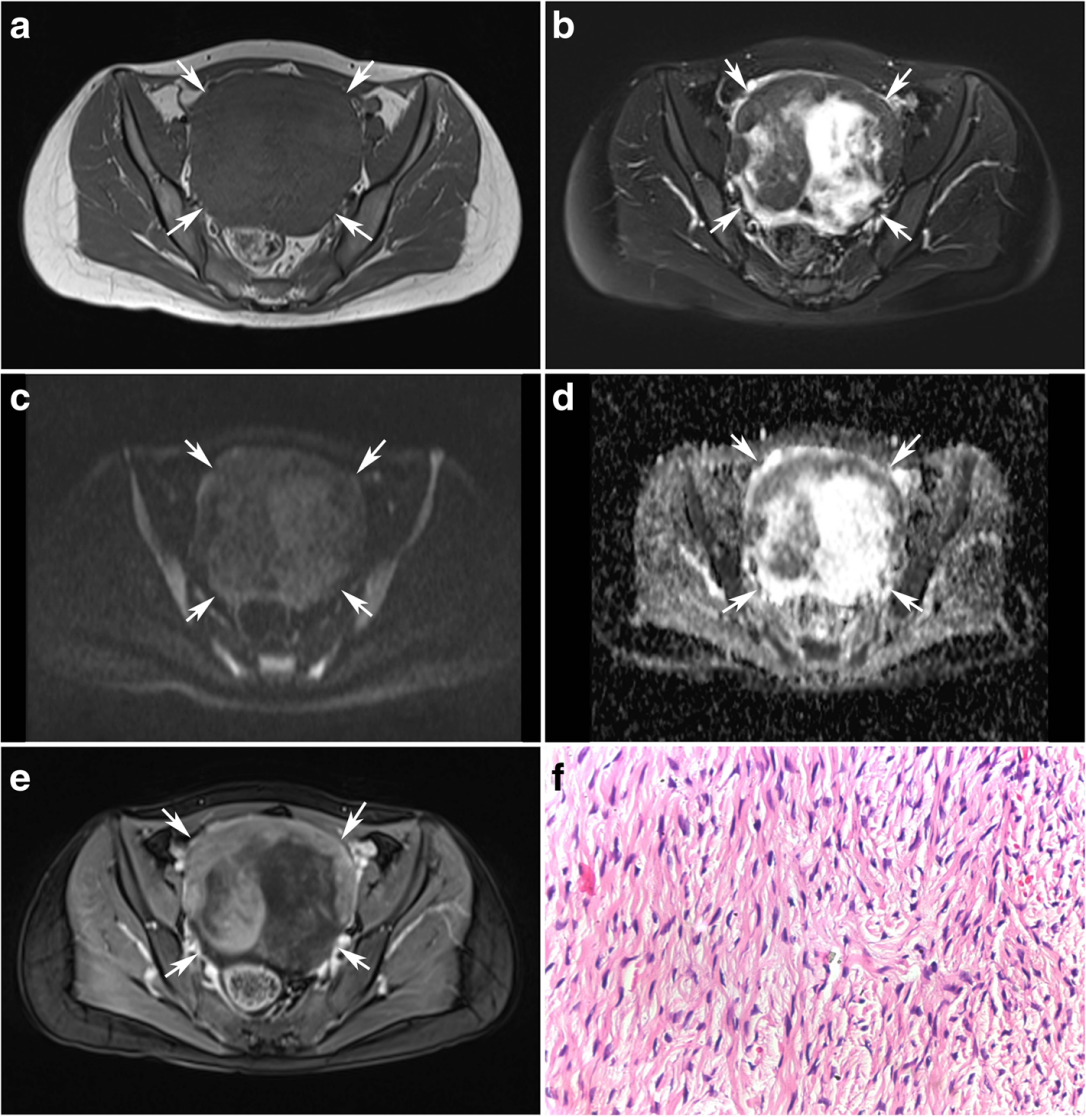
A 38-year-old women with a fibroma on the right ovary(arrows). Axial T1-weighted image (**a**) shows an oval mass of low signal intensity. T2-weighted with fat suppression image (**b**) shows the mass has significant low signal intensity with edema area of high signal intensity. The mass shows low signal intensity on DW imaging (**c**) and has a significant low ADC value of 0.181 × 10^− 3^ mm^2^/s in the non-edema area (**d**). T1-weighted contrast enhanced images (**e**) shows the mass was slightly enhanced after delay. The photomicrograph (H&E, × 400) (**f**) shows abundant fibrocytes

T2 signal intensity of the 42 ovarian sex cord-stromal tumors are summarized in Table 2. Malignant SCSTs showed higher T2 SI than benign SCSTs (Wilcoxon rank sum test, *p* = 0.04). T2 hypointensity was only seen in benign SCSTs with a sensitivity of 58.6% and specificity of 92.3% in diagnosing benign SCSTs which yield an accuracy of 69.0% for differential diagnosis of the two groups. All of the 10 fibromas were T2 hypointensity (Figs. 1 and 2).

**Table 2:** T2 signal intensity of the solid component of the 42 ovarian sex cord-stromal tumors

	Hypointensity	Isointensity	Hyperintensity
Benign	12	5	12
Purely stromal tumors			
Fibroma	10		
Cellular fibroma		1	1
Thecoma	1	1	5
Fibrothecoma	1	2	3
Sclerosing stromal tumor		1	3
Malignant	0	1	12
Purely sex cord tumors			
Granulosa cell tumor			7
Sex cord tumor with annular tubule			1
Mixed Sex cord-stromal tumors			
Sertoli-leydig tumor			4
Granulosathecoma		1

Contrast enhancement pattern of the 42 ovarian sex cord-stromal tumors are summarized in Table 3. Malignant SCSTs did not show more enhancement than benign SCSTs (Wilcoxon rank sum test, *p* >  0.05). However, mild enhancement was only seen in benign SCSTs with a sensitivity of 58.6% and specificity of 100% in diagnosing benign SCSTs which yield an accuracy of 71.4% for differential diagnosis of the two groups (Figs. 1 and 2).

**Table 3:** Contrast enhancement pattern of the 42 ovarian sex cord-stromal tumors

	Mild	Moderate	Intense
Benign	17	8	4
Purely stromal tumors			
Fibroma	9	1	
Cellular fibroma		2	
Thecoma	4	3	
Fibrothecoma	4	2	
Sclerosing stromal tumor			4
Malignant	0	9	4
Purely sex cord tumors			
Granulosa cell tumor		7	
Sex cord tumor with annular tubule		1	
Mixed Sex cord-stromal tumors			
Sertoli-leydig tumor			4
Granulosathecoma		1	

**Fig. 3:**
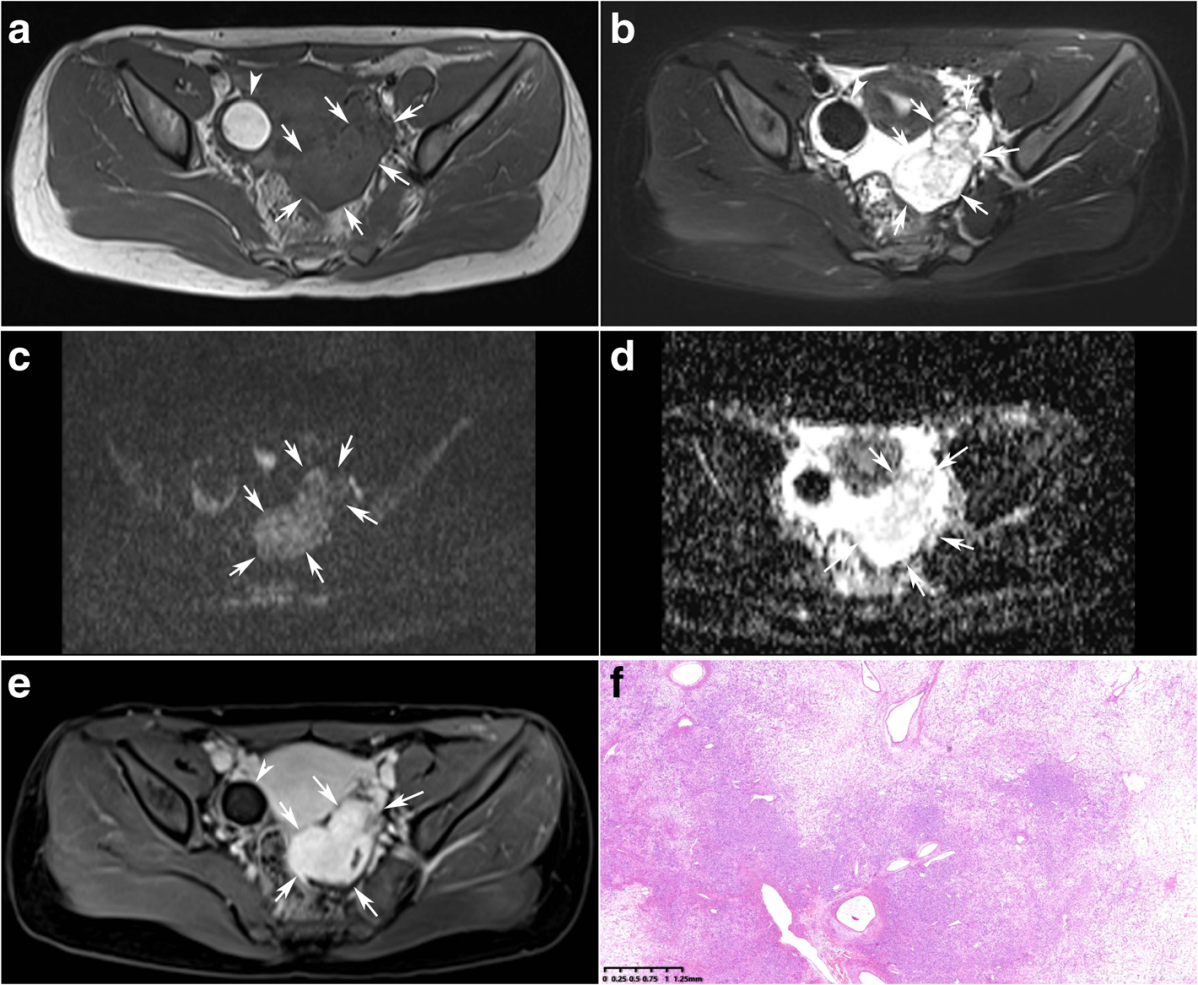
A 30-year-old young women with a sclerosing stromal tumor on the left ovary (arrows) combined with a mature teratoma on the right ovary(arrow heads). Axial T1-weighted image (**a**) shows a mass of low signal intensity. T2-weighted with fat suppression image (**b**) shows the mass has heterogenous high signal intensity. The mass shows moderate signal intensity on DW imaging (**c**) and has a significant high ADC value of 2.291 × 10^− 3^ mm^2^/s (**d**). T1-weighted contrast enhanced images (**e**) showed the mass was enhanced significantly. The photomicrograph (H&E, × 40) (**f**) shows the pseudolobulation of the cellular areas separated by hypocellular areas of loose edematous connective tissue

Fibromas were the tumors with the lowest observed ADC value (0.470 × 10^− 3^ mm^2^/s) (Table 1, Figs. 1 and 2). Sclerosing stromal tumors were the tumors with the highest observed ADC value (2.291 × 10^− 3^ mm^2^/s) (Table 1, Fig. 3).

**Fig. 4:**
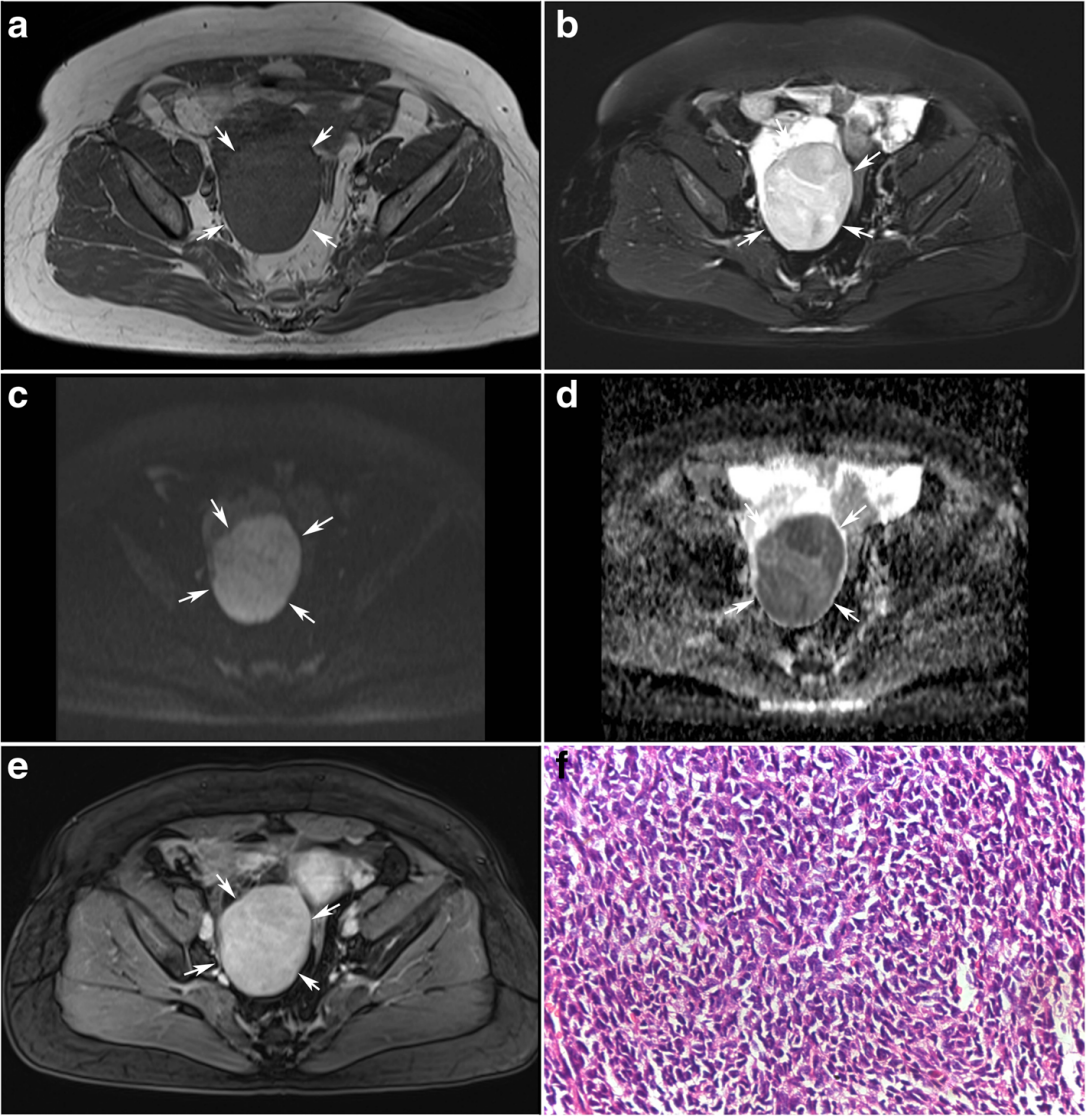
A 55-year women with an adult granulosa cell tumor on the right ovary(arrows). Axial T1-weighted image (**a**) shows a solid mass of low signal intensity. Axial T2-weighted with fat suppression images (**b**) show the mass has slightly high signal intensity. The solid mass shows high signal intensity on DW imaging (**c**) and has a low ADC value of 0.588 × 10^− 3^ mm^2^/s (**d**). T1-weighted contrast enhanced images (**e**) showed the solid mass was enhanced moderately. The photomicrograph (H&E, × 400) (**f**) confirmed the diagnosis of an adult granulosa cell tumor

DW imaging findings of the 42 ovarian sex cord-stromal tumors are summarized in Table 4. The solid component showed high SI in 12 of 13 (92.3%) malignant SCSTs versus in 3 of 29 (10.3%) benign SCSTs (*p* = 0.000) (Fig. 4).

**Table 4:** DW imaging signal intensity (SI) and ADC value of benign and malignant ovarian sex cord-stromal tumors

	Benign(29)	Malignant(13)	*p* value
SI			< 0.001
Low	11	0	
Moderate	15	1	
High	3	12	
ADC value (×10^−3^ mm^2^/s)^a^	0.951 ± 0.625	0.825 ± 0.129	0.639
ADC value (×10^− 3^ mm^2^/s)^b^	1.343 ± 0.528	0.825 ± 0.129	0.024

^a^ADC values was measured and averaged in 29 benign SCSTs and 13 malignant SCSTs respectively;

^b^ADC values was measured and averaged in 19 benign SCSTs (fibromas were excluded) and 13 malignant

SCSTs respectively

**Fig. 5:**
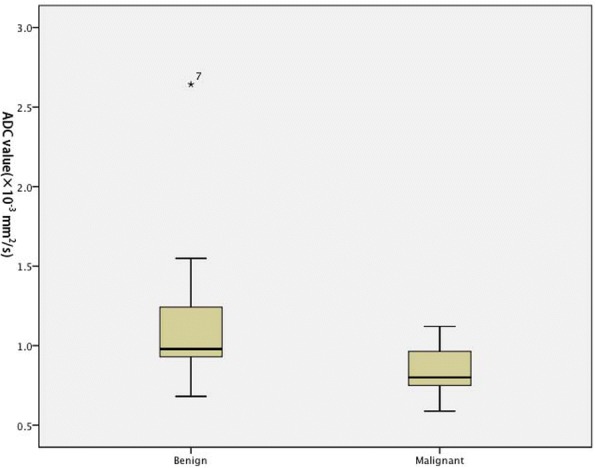
Boxplot of the ADC value of sex cord-stromal tumor of the ovary. ADC value of solid component was significantly lower in malignant SCSTs (0.825 ± 0.129 × 10^− 3^ mm^2^/s) than in benign SCSTs (1.343 ± 0.528 × 10^− 3^ mm^2^/s) when fibromas were excluded

There was no significant difference in ADC values of solid component between the two groups when fibromas were included (*p* = 0.639). However, ADC value of solid component was significantly lower in malignant SCSTs (0.825 ± 0.129 × 10^− 3^ mm^2^/s) than in benign SCSTs (1.343 ± 0.528 × 10^− 3^ mm^2^/s) when fibromas were excluded (*p* = 0.024) (Figs. 4 and 5). ROC curve analysis yielded an optimal ADC value threshold of 0.838 × 10^− 3^ mm^2^/s for differentiating malignant from benign tumors with a sensitivity of 61.5%, a specificity of 89.5% and an accuracy of 78.1%.

Considering each of the three sequences of T2, DCE and DW imaging has a limited value on the differential diagnosis of the benign and malignant SCSTs with an accuracy of 69.0%,71.4% and 78.1% respectively, we developed a multiparametric assessment which combined T2 with DCE and DW imaging. The assessment uses a 3-point scale based on the likelihood that the MRI findings correlates with the malignance of an ovarian sex cord-stromal tumor (Table 5). The assessment of the ovarian sex cord-stromal tumors was categorized as: 1- benign; 2- high likelihood to be benign; 3- high likelihood to be malignant. Category 1 (benign) was defined as a lesion which has typical MRI findings of T2 hypointensity or mild enhancement (Figs. 1 and 2). In case DCE showed non-mild enhancement, the lesion is further assessed on DW imaging. Category 2 (high likelihood to be benign) was define as a lesion which has non-mild enhancement with a high ADC value(Fig. 3). When a lesion showed moderate enhancement, a lower ADC value than 0.838 × 10^− 3^ mm^2^/s uprate the lesion to category 3(high likelihood to be malignant) (Fig. 4). When a lesion showed intense enhancement, a lower ADC value than 1.000 × 10^− 3^ mm^2^/s uprate the lesion to category 3(high likelihood to be malignant).

**Table 5:** Assessment of the ovarian sex cord-stromal tumors on a 3-point scale based on combination of T2 signal intensity, contrast enhancement pattern and ADC value

	T2	Enhancement	ADC value
1	hypointensity	–	–
	–	mild	
2	–	moderate	> 0.838 × 10^− 3^ mm^2^/s
		intense	> 1.000 × 10^−3^ mm^2^/s
3	–	moderate	≤ 0.838 × 10^−3^ mm^2^/s
		intense	≤ 1.000 × 10^−3^ mm^2^/s

Combination of T2, DCE and DW imaging is helpful for differential diagnosis of benign and malignant SCSTs with an accuracy of 88.0%.

Degeneration in SCSTs demonstrated as edema, hemorrhage and demarcated cysts (Table 6).

**Table 6:** Comparison of degeneration type and frequency between benign and malignant ovarian sex cord-stromal tumors

Degeneration	Benign(29)	Malignant(13)	*p* value
Edema	12 (41%)	0	< 0.001
Hemorrhage	4 (14%)	0	< 0.001
Demarcated cysts	4 (14%)	12 (90%)	0.024

Edema and hemorrhage was only found in benign SCSTs. Edema area in lesion showed moderate signal intensity on DW imaging with a high ADC value. Hemorrhage showed high signal intensity on DW imaging with a low ADC value. Demarcated cysts were found in 14% of benign lesions versus 90% of malignant lesions. Numerous mini cysts within the solid mass were only seen in malignant lesions.

## Discussion

Fibromas can be distinguished from other types of SCSTs based on the specific features on conventional MR imaging. Shinagare [14] reported that fibromas are abundant in bland spindle cells and intercellular collagen which contribute to the remarkable low signal intensity on T2WI and slight enhancement by contrast agent [15–18]. In this study, we found that T2 hypointensity is more specific than mild enhancement to fibromas. So T2WI is the dominant sequence to distinguish fibromas from the the rest of SCSTs. Also, fibromas were the tumors with the lowest ADC value. Bland spindle cells and intercellular collagen lined so densely that water molecule movement is more restricted in less intercellular space.

Although both T2 hypointensity and mild enhancement play a role in distinguishing benign SCSTs with a specificity of 100%, mild enhancement is more sensitive to benign SCSTs (Category 1). In case a lesion has a non-mild enhancement, the lesion should be further assessed on DW imaging (Category 2). The ADC value threshold to uprate a lesion of Category 2 to Category 3 was 0.838 × 10^− 3^ mm^2^/s.

Sclerosing stromal tumors were the only subtype of benign SCSTs which were enhanced intensely [2, 3]. We found that sclerosing stromal tumors were the tumors with the highest observed ADC value (2.291 × 10^− 3^ mm^2^/s). The explanation may be that the tumor has a characteristic microscopic pattern with pseudolobulation of the cellular areas separated by hypocellular areas of loose edematous connective tissue. Atram [19] reported that the edematous stromal change is a constant feature of sclerosing stromal tumors of the ovary. Sertoli-leydig tumors were the subtype of malignant SCSTs which were enhanced intensely [2, 3, 20]. The mean ADC value of sertoli-leydig tumors was 1.009 × 10^− 3^ mm^2^/s. So when a lesion showed intensely enhancement, we recommended a much higher ADC value threshold of 1.000 × 10^− 3^ mm^2^/s to uprate the lesion to Category 3.

Degeneration in SCSTs is common which demonstrated as edema, hemorrhage and cyst change [14, 21]. Edema and hemorrhage were only observed in benign lesions. However, demarcated cysts were observed in benign and malignant lesions. Kato [21] reported that up to 53% of fibromas demonstrated intratumoral cysts in their study.

There are some limitations in our study. First, it is a retrospective study. We just try to discuss about differentiation between benign and malignant sex cord-stromal tumors. We did not discuss about how to differentiate SCSTs from other pathological types of ovarian solid tumors. Prospective large sample investigation merits further study. Secondly, completely objective measurements of ADC value are usually difficult because sex cord-stromal tumors tumors tend to be heterogeneous masses. Thirdly, the variation in ADC between the subtypes of sex cord-stromal tumors is large.

## Conclusions

T2, DCE and DW imaging has a limited value on the differential diagnosis of the benign and malignant SCSTs with an accuracy of 69.0%,71.4% and 78.1% respectively. Combination of T2, DCE and DW imaging is more helpful which permit the distinction with an accuracy of 88.0%.

## Abbreviations

ADC: Apparent diffusion coefficient
DW: Diffusion-weighted
MR: magnetic resonance
SCST: Sex cord-stromal tumors of the ovary
